# A Model of Cell Biological Signaling Predicts a Phase Transition of Signaling and Provides Mathematical Formulae

**DOI:** 10.1371/journal.pone.0102911

**Published:** 2014-07-31

**Authors:** Tatsuaki Tsuruyama

**Affiliations:** Department of Molecular Pathology, Kyoto University Graduate School of Medicine, Kyoto city, Kyoto Prefecture, Japan; University of Georgia, United States of America

## Abstract

A biological signal is transmitted by interactions between signaling molecules in the cell. To date, there have been extensive studies regarding signaling pathways using numerical simulation of kinetic equations that are based on equations of continuity and Fick’s law. To obtain a mathematical formulation of cell signaling, we propose a stability kinetic model of cell biological signaling of a simple two-parameter model based on the kinetics of the diffusion-limiting step. In the present model, the signaling is regulated by the binding of a cofactor, such as ATP. Non-linearity of the kinetics is given by the diffusion fluctuation in the interaction between signaling molecules, which is different from previous works that hypothesized autocatalytic reactions. Numerical simulations showed the presence of a critical concentration of the cofactor beyond which the cell signaling molecule concentration is altered in a chaos-like oscillation with frequency, which is similar to a discontinuous phase transition in physics. Notably, we found that the frequency is given by the logarithm function of the difference of the outside cofactor concentration from the critical concentration. This implies that the outside alteration of the cofactor concentration is transformed into the oscillatory alteration of cell inner signaling. Further, mathematical stability kinetic analysis predicted a discontinuous dynamic phase transition in the critical state at which the cofactor concentration is equivalent to the critical concentration. In conclusion, the present model illustrates a unique feature of cell signaling, and the stability analysis may provide an analytical framework of the cell signaling system and a novel formulation of biological signaling.

## Introduction

Protein interaction is essential for cellular activities such as cytoskeleton formation [1], [2], protein assembly [3], and cellular signaling [4]. The MAPK signal pathway is an example of a cellular signaling pathway that has been extensively studied [5]–[8]. A transient binding cofactor such as ATP/GTP, or phosphorylation of amino acid residues, controls signaling molecule interactions and the subsequent modification of signaling molecules. Resulting reaction cascades operate to transmit cellular signals [6]. In cell signaling, oscillation has been reported in many studies, with circadian rhythms being a well-known example [9]. Calcium ion signaling oscillation is another well-known phenomenon [10]. Mathematical models have been proposed to explain signaling kinetic behaviors based on a set of kinetic equations. Systems biology approaches have also been developed in recent years [1], [2], [11].

Systems biology can describe the kinetics of a signal pathway usingsimultaneousequations of a complex reaction network. On the other hand, there are other types of models consisting of many simultaneous reaction rate equations, including more than ten variables [12], [13]; furthermore, systems biology models including more variables are also known [14]. Signaling networks frequently include non-linear reaction autocatalytic processes. To date, there have been many fascinating models of such autocatalytic reactions, enabling bifurcation and/or bi-stability in association with physical theory [5], [15]. However, autocatalytic models or positive feedback are not necessarily applicable to all biological signaling pathways.

In the current study, to understand biological signaling pathways, we used the following three novel perspectives (A)–(C) based on a non-linear and non-equilibrium kinetic model, which included only two concentrations of the signaling molecule to describe the biological signaling pathway. (A) An equation of the continuity of the chemical concentration of *c_i_* (*i* = *1…., n*) including chemical reaction items can be described using diffusion coefficients *D_i_*, kinetic coefficients *k_i_*, and concentrations of individual compounds *c_i_* as follows:
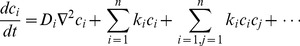
(1.1)


In the above formula, the diffusion rate is hypothesized to obey Fick’s law. In general, the diffusion items and chemical reaction items are thus separately described. On the other hand, because the biological signaling pathway network, including protein interactions, is limited by the slow diffusion rate of the signaling molecular proteins, kinetic coefficients generally depend on the diffusion coefficient. Therefore, diffusion items and reaction items cannot necessarily be separated without validation in the biological reaction. In the current model, we therefore described kinetic coefficients in a diffusion-coefficient-dependent manner.

(B) A feedback process due to non-linear self-catalytic reactions was not assumed in the current model, but instead, interactions between signaling molecules in their diffusion was assumed to give non-linearity to the model.

(C) A model system far from equilibrium due to a continuous supply of chemicals from the outside was hypothesized. The main issue is how minimal extracellular changes can be transformed into intracellular environmental changes. We aimed to evaluate the behavior of the model around the critical state by perturbation expansion using a minimal change of the supplied molecule concentration. By this mathematical evaluation, we aimed to illustrate the dynamic continuous oscillatory concentration change of signaling molecules from a static state.

In the current study, we constructed a novel model and aimed to evaluate the general intrinsic properties underlying cellular signaling based on signaling molecule interaction kinetics. Previous systems biology models have not necessarily focused on the diffusion process of proteins. Given the non-linearity during diffusion, we assumed kinetic instability of the signaling molecule interaction, and the sensitivity of the cell signaling in response to the environmental change was evaluated. The model system consists of several steps as follows: (i) the signaling molecule achieves an interaction active state by reversibly binding a cofactor that provides the signaling molecule with interaction activity; (ii) the signaling molecule has the ability to hydrolyze the cofactor; (iii) the signaling molecule interaction activity becomes lower when binding a hydrolyzed inactive cofactor compared to the signaling molecule binding an active cofactor; (iv) the signaling molecule has the ability to exchange the inactive cofactor with an active one; (v) active cofactors are supplied continuously from the outside. Thus, we set the interaction activity to be self-limiting, causing dynamic instability of the signaling molecule interaction. In the present model, we assumed that a signaling protein diffuses relatively slowly in the cytoplasm, and the whole signal transduction is a diffusion-limited reaction (assumption **A.1**). Following the protein interaction, one of the signaling molecules is phosphorylated or is bound to a cofactor such as GTP or ATP. In the kinetics of the protein interaction, fluctuation analysis was not fully performed in spite of the greater fluctuation in concentration relative to the solution of small molecules. Furthermore, we systematically analyzed the roles of the fluctuation in cellular signaling.

## Materials and Methods

### Numerical simulation

Numerical calculations were performed using Mathematica 8 (Wolfram Research, Inc., Champaign, IL).

## Results

### Protein interaction kinetics

**Figure 1:**
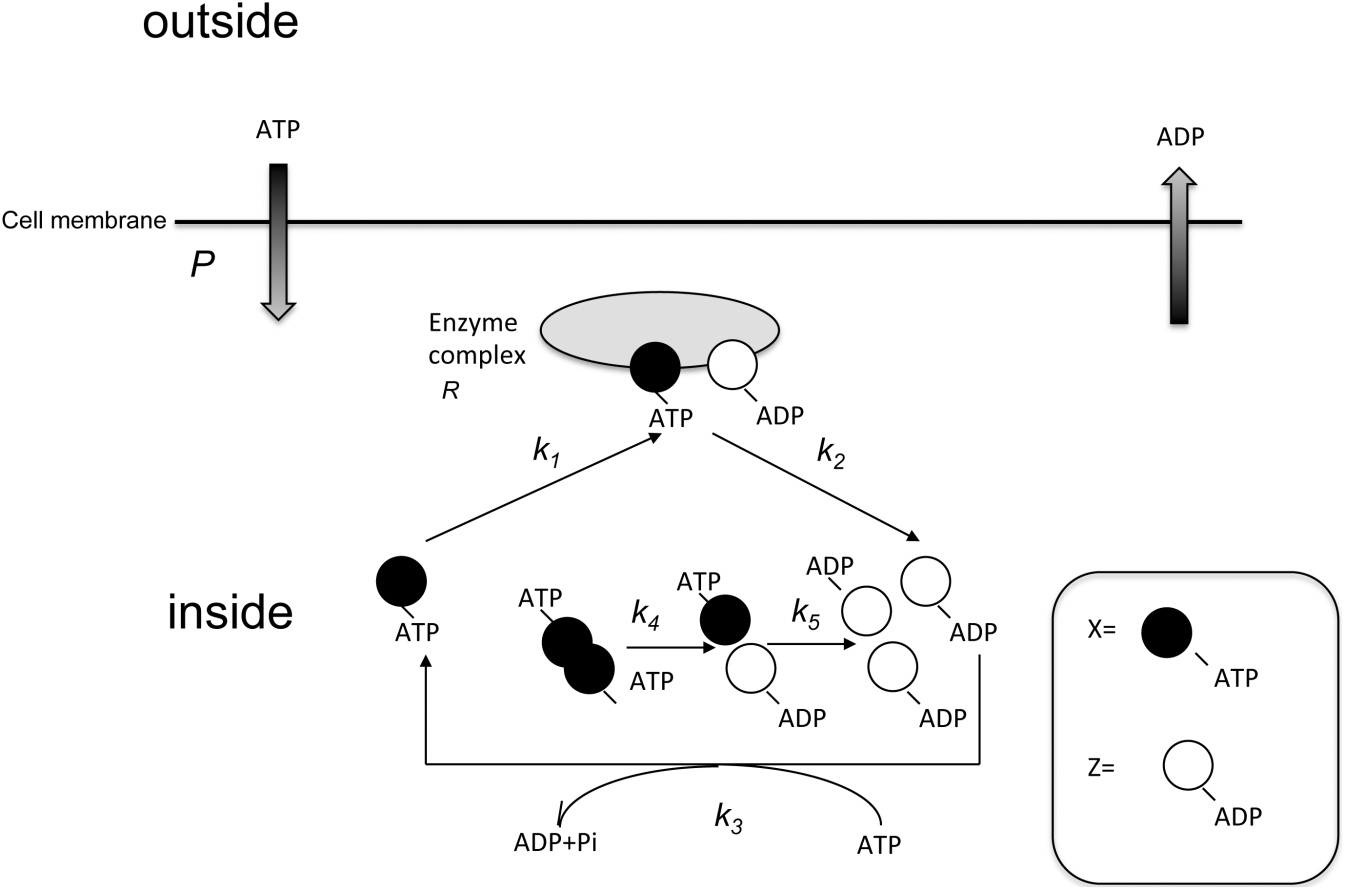
Scheme of signaling molecule interaction. Individual globules or oblongs represent signaling molecules *X, Y, Z*, and receptor *R*. Kinetic coefficients are shown next to the arrows. Outside and inside signify the outside and the inside of the cell, respectively.



The model scheme is shown in Figure 1. There are two types of signaling molecule, an active cofactor-binding signaling molecule (*X*), and an inactive cofactor-binding signaling molecule (*Z*). An active cofactor is non-hydrolyzed, and the inactive cofactor is the hydrolyzed type. *X* has the higher interactive activity and *Z* has the lower interactive activity. First, *X* can associate with oligomeric enzyme complex *R* consisting of *X* and *Z*, which transforms the active form *X* into the inactive form *Z* by hydrolysis of the binding cofactor and is to be released as *Z* irreversibly:

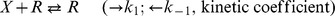


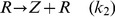
(2.1)


In the above formula, we assumed that signaling proteins diffuse relatively slowly in the cytoplasm and that the dissociation rate (relating to the irreversible orientation *k_−1_*) of an encountering pair *X* and *Z* is significantly slower than the hydrolysis rate of the active cofactor changing into inactive cofactor (relating to *k_2_*) (assumption 2; **A.2**). On a simple consideration of the diffusion limited step, when the kinetic rate can be described according to Fick’s law using diffusion coefficients *D_X_* and *D_Z_* of *X* and *Z*, respectively:
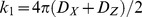
(2.2)


Next, *Z* recovers its interaction activity by exchange active cofactor *P* into inactive cofactor *P’*, returning to *X* (Figure 1).
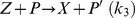
(2.3)


Signaling molecules have the potential to hydrolyze the cofactor by interacting with identical species:
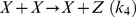
(2.4)

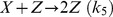
(2.5)


In (2.4) and (2.5), likewise in (1.2):
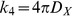
(2.6)

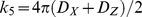
(2.7)


### Kinetic equations of interaction signaling molecules

Here, kinetic equations were set according to the above simple reaction cascade. The equations consist of protein interactive items and an item of small-molecule-cofactor exchange. When the concentration of the protein is sufficiently small, the dependency of the diffusion coefficient on the concentration is linear [11], [13]–[18]. In comparison with the exchange kinetic rate of the cofactor (2.3), the rate of macromolecular protein interaction that depends on the diffusion step can be regarded as significantly smaller ((1.1), (2.4), & (2.5)) as a general (assumption 1; **A.1**). In this case, the whole reaction system can be regarded as diffusion-limited [4], [19], [20], and the diffusion rate is given according to Fick’s law using a gradient of concentration. Thus, we get the protein interaction kinetics equations using the diffusion coefficient:
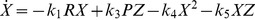
(2.8)


Here, the exchange rate of the cofactor is expressed by the item *k_3_* ’ *PZ*. Further,
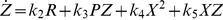
(2.9)


Because kinetic coefficients depend on diffusion coefficients, we set:
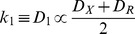


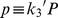


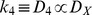


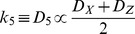
(2.10)


Using (2.10), (2.8) and (2.9) are rewritten as follows:
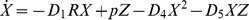
(2.11)

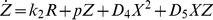
(2.12)


Here we set the oligomer concentration as constant because *de novo* asssembly is considered to be much slower than monomer interaction at the steady state (assumption 3; **A.3**):
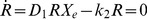
(2.13)


Here, a small letter affixed signifies values at the steady state.

This assumption is based upon on the steady state in the protein assembly [21]–[24].

Setting the right hand sides of Eqs. (2.11) and (2.12) gives:
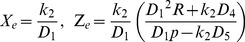
(2.14)


### Fluctuation of diffusion coefficient

Subsequently, let us consider the fluctuation of participant proteins. We set:
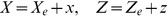
(3.1)


In actuality, receptor *R* interacts with other proteins, *X* and *Z*, in the course of diffusion (Figure 1). In actual signaling pathways, signaling molecules associate with other signaling molecules and phosphorylate them or are phosphorylated by them. The diffusion coefficients can be altered in proportion to the signaling molecule concentration. By using the Gibbs-Duhem expression, the diffusion coefficient *D* of one macromolecule in the solution can be generally written as [13]:
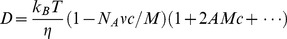
(3.2)where *T* is the temperature of the solution, *k_B_* is the Boltzmann constant, and *η* is the frictional coefficient of a given macromolecule in solution. A is the second virial coefficient, *v* is the partial specific volume of protein with molecular weight *M*, and *N_A_* is Avogadro’s number. The small letter *c* denotes the concentration of the solute.

Further, we hypothesized that *D_R_*, *D_X_,* and *D_Z_,* the diffusion coefficients of *R*, *X* and *Z*, are given by extension of (3.2) to the mix solution of two macromolecules, *X* and *Z:*

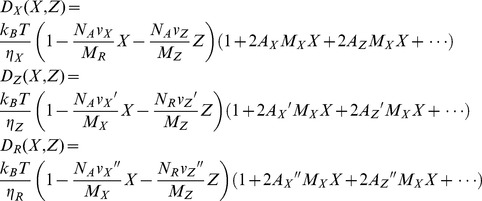
(3.3)where *v_X_* and *v_Z_* are the partial specific volumes of *X* and *Z* with molecular weights *M_X_* and *M_Z_*, respectively. *A_X_ A_X_’, A_X_’’, A_Z_*, *A_Z_’*, and *A_Z_’’* are the second virial coefficients. In actuality, *X* and *Z* are the same molecules except with bound ATP or ADP. The fluctuation of the diffusion coefficient is given by:



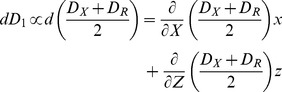
(3.4)And therefore, we set:
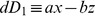
(3.5)


Here, an increase in *X* contributes to a decrease in diffusion coefficient *D_1_* in thefluctuation item, *ax* (*a*>0), because of the higher interaction activity that reducesdiffusion; in contrast, increased *Z* contributes to increases in the diffusion coefficient *D_1_* in the fluctuation item *bz* (*b*>0).

Likewise in (4.5),
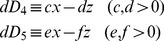
(3.6)


Using (3.5) and (3.6), Eqs. (2.5) and (2.7) give the fluctuation kinetic equations:
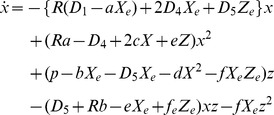
(3.7)

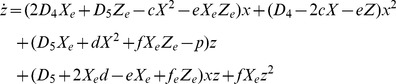
(3.8)


Further, using matrix formulation,
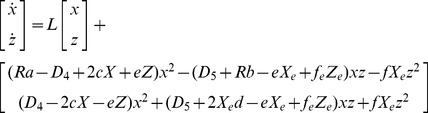
(3.9)


Here,
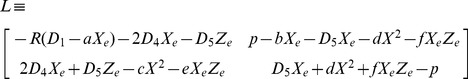
(3.10)


### Calculus simulation of concentration oscillation

**Figure 2:**
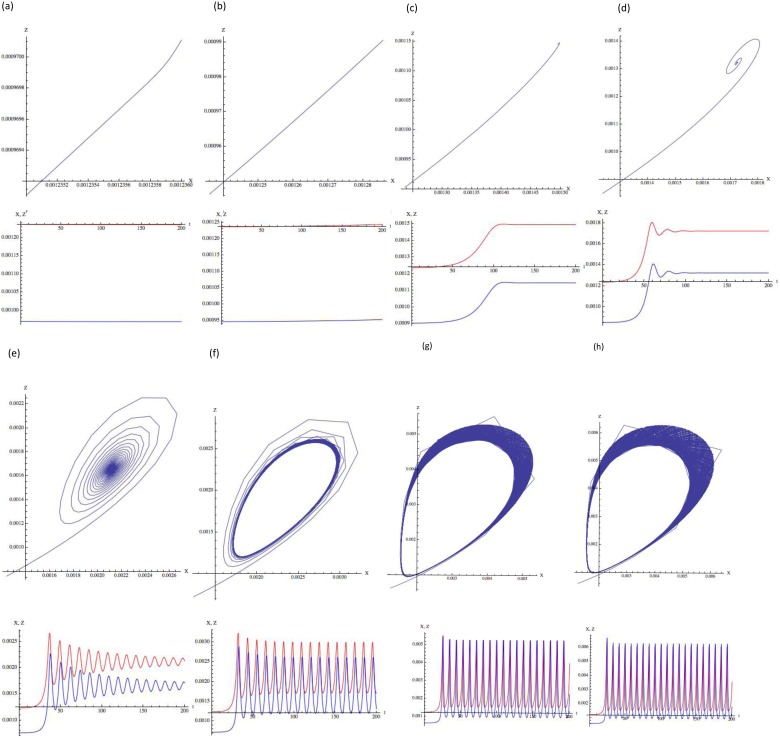
Time-course of the fluctuation of the signaling molecules displays a chaos-like oscillation. Diffusion of active cofactor binding signaling molecule (*X*) and of inactive cofactor binding signaling molecule (*Z*). The Appendix S1 presents the simulation parameters, with the notation of Eqs. (3.9). *p* is (a) 0.795, (b) 0.81, (c) 0.84, (d) 0.88, (e) 0.96, (f) 1.00, (g) 1.12, and (h) 1.16. The upper graph shows two parametric plots of *X,* and *Z*. Red, and blue lines in the lower graph represent the concentrations of *X*, and *Z*, respectively. The horizontal axis represents time (0 ≤ *t ≤* 200) and the vertical axis represents the concentrations of *X*, and *Z*, respectively. When *p* exceeds 0.80, chaos-like oscillation is observed. Mathematica cord when *p* = 0.795 (a) is shown below. Below is the simulation program when *p* = 1.0253: D1 = 0.28 k2 = 0.00034580 a = 800 b = 656 c = 100 d = 100 e = 100 f = 100 p = 1.0253 D4 = 156 D5 = 156 R = 1 X = k2/D1 Z = (k2 (D1∧2 R+ D4 k2))/(D1 (D1 p - D5 k2)) NDSolve[{Derivative [1] [x][t] =  = -(R (D1 - a X) +2 X D4+ D5 Z) x[t] + (R a - D4+2 c X + e Z) x[t]∧2+ (p - D5 X - b X - d X∧2 - f X Z) z[t] - (D5+ R b - e X + f Z) x[t] z[t] - (f X) z[t]∧2, Derivative [1] [z][t] =  = (2 X D4+ D5 Z - c X∧2 - e X Z) x[t] + (D4–2 c X - e Z) x[t]∧2+ (D5+2 X d - e X + f Z) x[t] z[t] + (D5 X - p + d X∧2+ f X Z) z[t], x[0] =  = 1.’*∧-6, z[0] =  = 1.’*∧-6}, {x, z}, {t, 0, 30000}, MaxSteps ->50000] g001 = Plot[{X + x[t]}/. %, {t, 0, 200}, PlotRange -> All, PlotStyle -> {RGBColor[1, 0, 0]}, PlotRange -> ALL] g003 = Plot[{Z + z[t]}/. %%, {t, 0, 200}, PlotRange -> All, PlotStyle -> {RGBColor[0, 0, 1]}, PlotRange -> All] g004 = ParametricPlot[Evaluate[{X + x[t], Z + z[t]}/. %%%], {t, 0, 2000}, PlotRange -> All, AxesLabel -> {"X", "Z"}] Show [g001, g003, AxesLabel -> {"t", "X, Z"}]



The time-course of the signaling molecule concentrations was simulated via the substitution of appropriate numerical values into (3.9). A numerical calculation was performed over a sufficiently long period to evaluate the trend of signaling protein behavior. In the current simulation, the fluctuation coefficients *a, b, c, d, e*, and *f*, of *D_1_*, *D_4_*, and *D_5_* are of the same order of magnitude, 10^2^ (100∼856). The concentrations of *X* and *Z* at the steady state are given by Eq. (2.14). On the basis of **A.3**, *D_1_*, the diffusion coefficient of the assembling rate of *X* to *R,* is significantly smaller than *D_4_* and *D_5_,* which are diffusion coefficients of the assembling rate between *X* and *X,* and *X* and *Z*. Applying the above conditions, the simulation results are shown in Figure 2. When *p* increases to the values that satisfy:

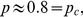
(4.1)the concentration of the signaling molecules continuously oscillates, showing nearly equivalent frequency and amplitude at oscillatory individual peaks, except for at the initial phase. Subsequently, we evaluated the mean amplitude and frequency of the fluctuation oscillation. From *t* = 50, around when the oscillation initiates, to *t* = 1000, the mean amplitude was calculated as the division of the sum of each size of the peak by the number of peaks. In addition, the frequency was estimated by dividing the number of the peak by *Δt* = 950.

**Figure 3:**
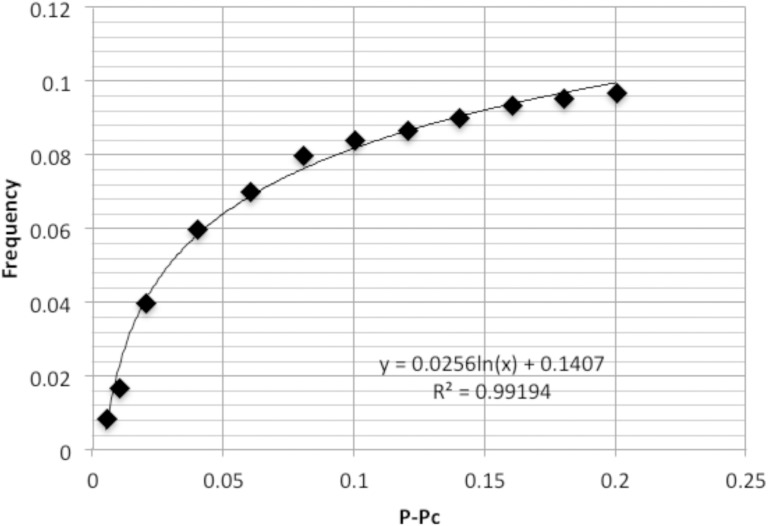
Plot of the mean amplitude and frequency. The frequency of the oscillation of fluctuation *x* is plotted in reference to the numerical calculation from ε = 0 to 0.25. The line is the result of regression analysis on logarithm function. The correlation coefficient is shown in the plot.



Notably, the present simulation shows that both frequency and amplitude are nearly proportional to the logarithm of ε = *p−p_c_* (Figure 3). Namely,

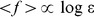
(4.2)These formulae imply that the outside alteration is transformed inside into the information of cell signaling. On the basis of the above simulation results, the present signaling model system is characterized by:
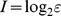



### Evaluation of the stability of the model around the equilibrium state

For mathematical analysis of stability around the critical point, Eq. (3.9) (4.10) was formulated. When *p* is equivalent to *p_c_* = 0.8, the matrix for (*x*, *z*) is given by **Lc** in (3.10):
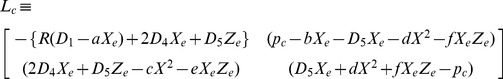
(5.1)


Using the eigenvectors of *L*, (***l_1_*** **,** ***l_2_***), coordinate transformation is performed as follows:
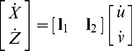
(6.2)

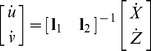
(6.3)


The above parameters are subsequently set as:
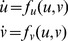
(6.4)


Further *u* is given by:
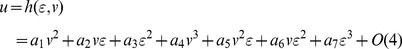
(6.5)


Therefore, using
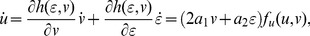
(6.6)we can then obtain using (6.5) and (6.6):



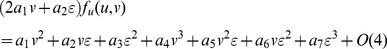
(6.7)Solving the above (6.7), the coefficients of *a_i_* in (6.5) are given (see Appendix S1: mathematica cord [#55–70]). By substitution of *u* that is given by *ν* and ε into *f_v_* (*u,v*) in (6.4), we can obtain the kinetic stability equation of fluctuation *ν* using coefficients *n_i_* (*i* = 1, 2, 3, 4, 5, 6) as follows (See Appendix S1, Out [#73] in the mathematica cord):
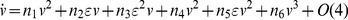
(6.8)


To evaluate the amplitude of fluctuation, setting the right hand equal to zero,
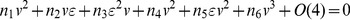
(6.9)


**Figure 4:**
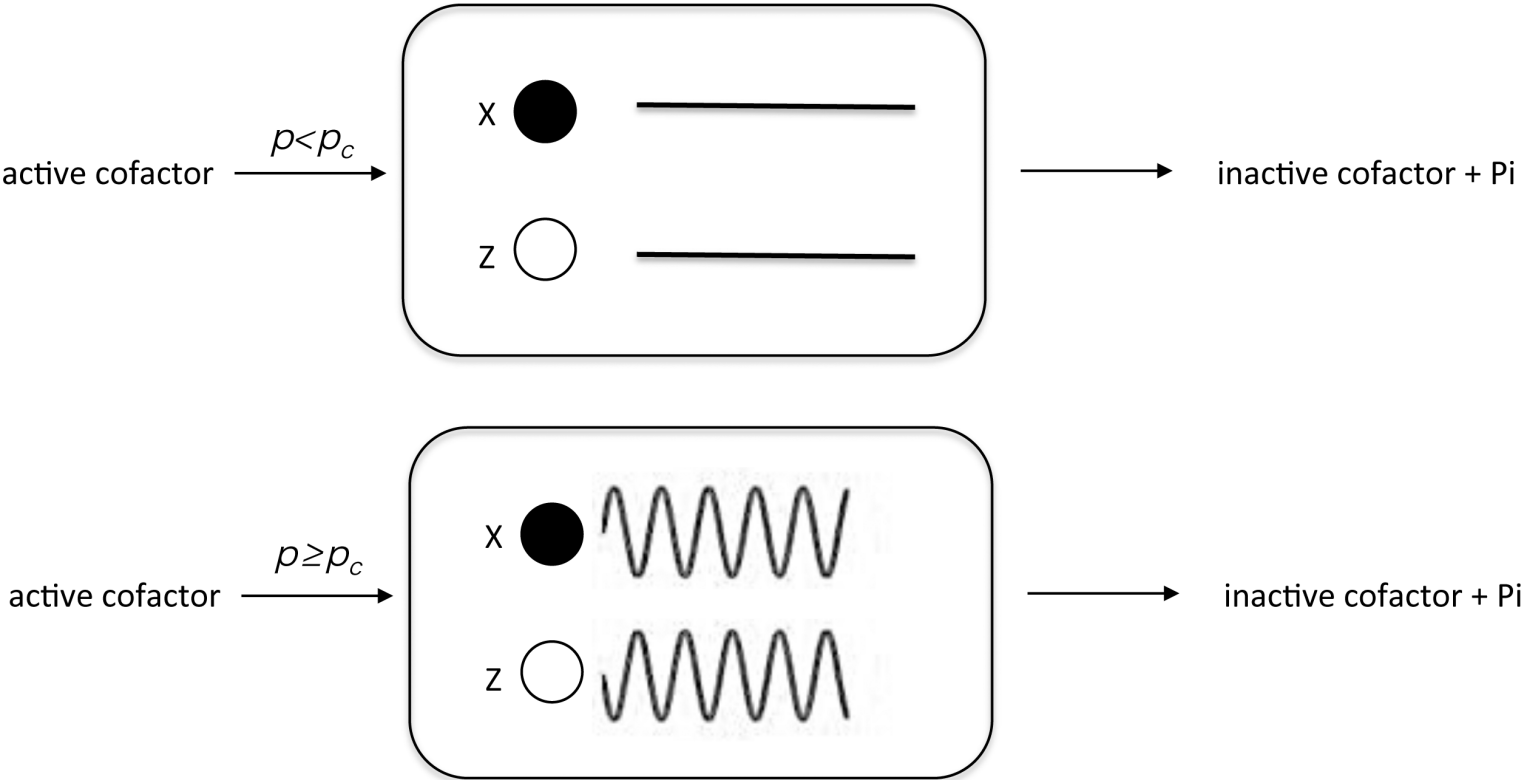
Scheme of the transformation of outside information into the intracellular signaling oscillation. The scheme illustrates the bifurcation of the fluctuation with respect to the increase in *p*.

As a result, when *ε*<7.0×10^−4^, Eq. (6.9) has two real number solutions of *v* other than zero, indicating the bi-stability of the fluctuation *v*; when *ε*≥7.0×10^−4^ (6.9), it has only the zero solution of the fluctuation (See Appendix S1; mathematica cord Out [#74]). Therefore, a bifurcation of the fluctuation with respect to the value of *ε* is predicted (Figure 4). Because *v* is simply given by the linear equation (6.3), this directly demonstrates the amplitude bifurcation of *x* and *z*.

## Discussion

Here, we presented a model of cell signaling systems and performed mathematical analysis on the model in addition to numerical simulations. An increase in the supply of the cofactor near the critical concentration induces a ‘phase transition’ of the system, indicating that the model system has the ability to transform information on the concentration change of a cofactor outside the system into inside information, i.e., the amplitude or frequency of the concentration oscillation of the signaling molecule. This term, ‘phase transition’, is a metaphor implying that the model system nearly discontinuously acquires the ability to dynamically transform outside information into inside information.

The introduced non-linear kinetic equations include only two independent parameters, the active or inactive cofactor binding protein. The observed oscillation of signaling molecules in the simulation is not a chaotic behavior that requires more than two parameters [25]. However, the fluctuation of the signaling molecule concentration shows chaos-like oscillatory behavior. In fact, neither the amplitude nor frequency of every oscillation is precisely constant for a lengthy period as shown in the trajectory. We will report mathematical validation of the result elsewhere. We will report mathematical validation of the result elsewhere.

The simulation allowed us to define the formula (5.3) mentioned above. The formula using the logarithmic function brings to light an important idea. The present simple model illustrates an essential property in which a system is relatively stable to the outside environment, because a minimal increase in the concentration of the cofactor is transformed into the logarithm of the concentration change inside the cell system. These features may be crucial in responding to transformations of the outside environment while minimizing the inside influence caused by outside alteration. Further, the present simple formulation is reminiscent of the definition of entropy in informatics. In conclusion, our model indicates that the non-linearity of a protein interaction theoretically gives an interesting cohesive behavior, such as an oscillation, of the signaling system leading to self-organization *in vivo*. Still, the theoretical basis of cell signaling systems for quantitative evaluation requires further formulation on the basis of experimental study.

## Supporting Information

Appendix S1 (DOCX)
