# Correction: A Model of Cell Biological Signaling Predicts a Phase Transition of Signaling and Provides Mathematical Formulae

**DOI:** 10.1371/journal.pone.0112993

**Published:** 2014-11-04

**Authors:** The PLOS ONE Staff

There are errors in the legend for Figure 2, “Time-course of the fluctuation of the signaling molecules displays a chaos-like oscillation.” Please view Figure 2 and its complete, correct legend here.

**Figure 2:**
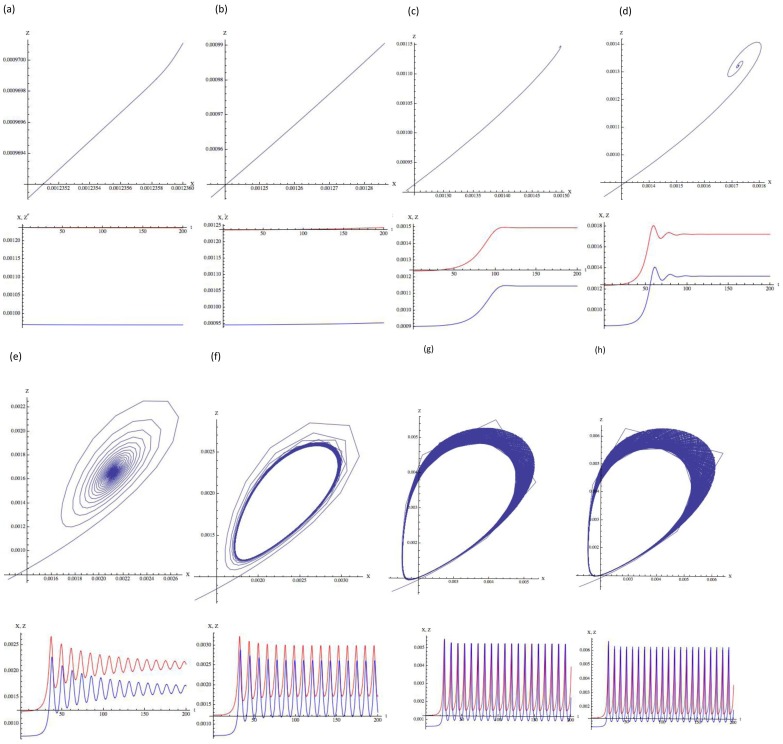
Time-course of the fluctuation of the signaling molecules displays a chaos-like oscillation. Diffusion of active cofactor binding signaling molecule (X) and of inactive cofactor binding signaling molecule (Z). The Appendix S1 presents the simulation parameters, with the notation of Eqs. (3.9). p is (a) 0.795, (b) 0.81, (c) 0.84, (d) 0.88, (e) 0.96, (f) 1.00, (g) 1.12, and (h) 1.16. The upper graph shows two parametric plots of X, and Z. Red, and blue lines in the lower graph represent the concentrations of X, and Z, respectively. The horizontal axis represents time (0 ≤ t ≤ 200) and the vertical axis represents the concentrations of X, and Z, respectively. When p exceeds 0.80, chaos-like oscillation is observed. Mathematica cord when p  =  0.795 (a) is shown below. Below is the simulation program when p  =  1.0253: D1  =  0.28 k2  =  0.00034580 a  =  800 b  =  656 c  =  100 d  =  100 e  =  100 f  =  100 p =  1.0523 D4  =  156 D5  =  156 R  =  1 X  =  k2/D1 Z  =  (k2 (D1∧2 R+ D4 k2))/(D1 (D1 p - D5 k2)) NDSolve[{Derivative[1][x][t]  =  =  -(R (D1 - a X) + 2 X D4 + D5 Z) x[t] + (R a - D4 + 2 c X + e Z) x[t]∧2 + (p - D5 X - b X - d X∧2 - f X Z) z[t] - (D5 + R b - e X + f Z) x[t] z[t] - (f X) z[t]∧2, Derivative[1][z][t]  =  =  (2 X D4 + D5 Z - c X∧2 - e X Z) x[t] + (D4 - 2 c X - e Z) x[t]∧2 + (D5 + 2 X d - e X + f Z) x[t] z[t] + (D5 X - p + d X∧2 + f X Z) z[t], x[0]  =  =  1.’*∧-6, z[0]  =  =  1.’*∧-6}, {x, z}, {t, 0, 30000}, MaxSteps -> 50000] g001  =  Plot[{X + x[t]}/. %, {t, 0, 200}, PlotRange -> All, PlotStyle -> {RGBColor[1, 0, 0]}, PlotRange -> ALL] g003  =  Plot[{Z + z[t]}/. %%, {t, 0, 200}, PlotRange -> All, PlotStyle -> {RGBColor[0, 0, 1]}, PlotRange -> All] g004  =  ParametricPlot[Evaluate[{X + x[t], Z + z[t]}/. %%%], {t, 0, 2000}, PlotRange -> All, AxesLabel -> {"X", "Z"}] Show [g001, g003, AxesLabel -> {"t", "X, Z"}].

In the Results, there is an error in last equation before the “Evaluation of the stability of the model around the equilibrium state” subsection. This equation should be numbered 4.3. Please view the complete, correct equation here: 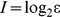
(4.3)


There is an error in the first sentence of the “Evaluation of the stability of the model around the equilibrium state” subsection of the Results. The correct sentence is: For mathematical analysis of stability around the critical point, Eq. (3.9) (4.3) was formulated.

There is an error in the first sentence of the last paragraph of the Discussion. The correct sentence is: The simulation allowed us to define the formula (4.3) mentioned above.
