# Supplementary material for: A Model of Cell Biological Signaling Predicts a Phase Transition of Signaling and Provides Mathematical Formulae
Source: PLoS One. 2014 Jul 31;9(7):e102911. doi: 10.1371/journal.pone.0102911 (PMC4117461; doi:10.1371/journal.pone.0102911)
Supplement: Appendix S1 — (DOCX) [file pone.0102911.s001.docx]

**Appendix**

**Mathematica codes for stability analysis around the critical state**

The center manifold of an equilibrium state of a dynamic system consists of orbits whose behavior around the equilibrium state is not controlled by either the attraction of the stable manifold or the repulsion of the unstable manifold. For linearization of the system, the eigenvectors of L corresponding to eigenvalues with negative real parts form the stable eigenspace, which gives rise to the stable manifold. Similarly, eigenvalues with positive real parts yield the unstable manifold. In this analysis, we set the Mathematica code as follows. Mathematica code input and output are expressed as In [#]: = and Out [#]:. The matrix L in (5.1) and the eigenvectors of L, (**l_1_, l_2_**), the transpose matrix of [**l_1_, l_2_**] in (5.2), and the inverse matrix [**l_1_, l_2_**]^-1^ in (5.3) are given by:

In [1]:

D1 = 0.28
k2 = 0.00034580
a = 800
b = 656
c = 100
d = 100
e = 100
f = 100
p = 1.0253
D4 = 156
D5 = 156
R = 1
X = k2/D1
Z = (k2 (D1^2 R+ D4 k2))/(D1 (D1 p - D5 k2))
L= [({{-(R (D1 - a X) + 2 X D4 + D5 Z), p - D5 X - b X - d X^2 - f X Z}, {2 X D4 + D5 Z - c X^2 - e X Z, D5 X - p + d X^2 + f X Z }})]

Eigenvectors [%]

Transpose [%]

Inverse [%]

> Out [12] gives the eigenvectors of L, (l_1_, l_2_). Out [16] gives transpose matrix of [l_1_, l_2_] in (5.2) and Out [14] gives the inverse matrix [l_1_, l_2_]^-1^ in (5.3):

Out[12]= {{0.335848, 0.941916}, {0.735928, 0.67706}}

Out[13]= {{0.335848, 0.735928}, {0.941916, 0.67706}}

Out[14]= {{-1.45356, 1.57995}, {2.02218, -0.721025}}

> To obtain in the left hand of (5.4) using Out [15.],

In[18]:= v' -> 2.0221770558212007` x' - 0.7210248928916749` z'
% /. x' -> -(R (D1 - a X) + 2 X D4 + D5 Z) x + (R a - D4 + 2 c X +
 g Z) x^2 + (p (1 + e) - D5 X - b X - d X^2 - f X Z) z - (D5 + R b - g X + f Z) x z - (f X) z^2
% /. z' -> (2 X D4 + D5 Z - c X^2 - g X Z) x + (D4 - 2 c X - g Z) x^2 + (D5 + 2 X d - g X + f Z) x z + (D5 X - p (1 + e) + d X^2 + f X Z) z
% /. x -> 0.335848396936556` u + 0.7359280914703337` v
% /. z -> 0.9419160547921165` u + 0.6770597050370315` v

> Then Out [22] gives (v’) :

Out[22] =v’ -> -0.721025 ((0.192932 - 0.7953 (1 + e)) (0.941916 u + 0.67706 v) +
 0.536154 (0.335848 u + 0.735928 v) +
 156.22 (0.941916 u + 0.67706 v) (0.335848 u + 0.735928 v) +
 155.656 (0.335848 u + 0.735928 v)^2) +
 2.02218 ((-1.00309 + 0.7953 (1 + e)) (0.941916 u + 0.67706 v) -
 0.1235 (0.941916 u + 0.67706 v)^2 + 0.171574 (0.335848 u + 0.735928 v) - 811.973 (0.941916 u + 0.67706 v) (0.335848 u + 0.735928 v) +
 644.344 (0.335848 u + 0.735928 v)^2)

> Here, we change the notation (v’) into v’’ in order to subsequent calculation:

In[23]: =v'' = -0.7210248928916749` ((0.1929321482879497` -
 0.7953` (1 + e)) (0.9419160547921165` u + 0.6770597050370315` v) + 0.5361541101748142` (0.335848396936556` u + 0.7359280914703337` v) + 156.2203629861941` (0.9419160547921165` u + 0.6770597050370315` v) (0.335848396936556` u + 0.7359280914703337` v) + 155.6561370138059` (0.335848396936556` u + 0.7359280914703337` v)^2) + 2.0221770558212007` ((-1.0030921482879498` + 0.7953` (1 + e)) (0.9419160547921165` u + 0.6770597050370315` v) - 0.1235` (0.9419160547921165` u + 0.6770597050370315` v)^2 + 0.17157374153723617` (0.335848396936556` u + 0.7359280914703337` v) - 811.973362986194` (0.9419160547921165` u + 0.6770597050370315` v) (0.335848396936556` u + 0.7359280914703337` v) + 644.343862986194` (0.335848396936556` u + 0.7359280914703337` v)^2)

>Subsequently, to obtain (*u’*) in (5.4) we set following mathematica code:

In[24]:= u' -> -1.453563291528274` x' + 1.5799464227563669` z'
% /. x' -> -(R (D1 - a X) + 2 X D4 + D5 Z) x + (R a - D4 + 2 c X +
 g Z) x^2 + (p (1 + e) - D5 X - b X - d X^2 - f X Z) z - (D5 + R b - g X + f Z) x z - (f X) z^2
% /. z' -> (2 X D4 + D5 Z - c X^2 - g X Z) x + (D4 - 2 c X - g Z) x^2 + (D5 + 2 X d - g X + f Z) x z + (D5 X - p (1 + e) + d X^2 + f X Z) z
% /. x -> 0.335848396936556` u + 0.7359280914703337` v
% /. z -> 0.9419160547921165` u + 0.6770597050370315` v

>The first input refers to the left component of {-1.35368, 1.51433}, in Out [17] for [l_1_, l_2_]^-1.^

Out[28]=u' -> 1.57995 ((0.192932 - 0.7953 (1 + e)) (0.941916 u + 0.67706 v) + 0.536154 (0.335848 u + 0.735928 v) + 156.22 (0.941916 u + 0.67706 v) (0.335848 u + 0.735928 v) + 155.656 (0.335848 u + 0.735928 v)^2) - 1.45356 ((-1.00309 + 0.7953 (1 + e)) (0.941916 u + 0.67706 v) - 0.1235 (0.941916 u + 0.67706 v)^2 + 0.171574 (0.335848 u + 0.735928 v) - 811.973 (0.941916 u + 0.67706 v) (0.335848 u + 0.735928 v) + 644.344 (0.335848 u + 0.735928 v)^2)

>Here we change the notation u’ into u’’ in order to subsequent calculation:

In[29]:= u'' = 1.5799464227563669` ((0.1929321482879497` - 0.7953` (1 + e)) (0.9419160547921165` u + 0.6770597050370315` v) + 0.5361541101748142` (0.335848396936556` u + 0.7359280914703337` v) + 156.2203629861941` (0.9419160547921165` u + 0.6770597050370315` v) (0.335848396936556` u + 0.7359280914703337` v) + 155.6561370138059` (0.335848396936556` u + 0.7359280914703337` v)^2) - 1.453563291528274` ((-1.0030921482879498` + 0.7953` (1 + e)) (0.9419160547921165` u + 0.6770597050370315` v) - 0.1235` (0.9419160547921165` u + 0.6770597050370315` v)^2 + 0.17157374153723617` (0.335848396936556` u + 0.7359280914703337` v) - 811.973362986194` (0.9419160547921165` u + 0.6770597050370315` v) (0.335848396936556` u + 0.7359280914703337` v) + 644.343862986194` (0.335848396936556` u + 0.7359280914703337` v)^2)

>Subsequently, we set referring to the right hand of.6),

In[30]:=u''' = (2 a1 v + a2 ε)*v''

In[31]:=u''' - u''

% /. u -> a1 v^2 + a2 v ε + a3 ε^2 + a4 v^3 + a5 ε v^2 + a6 v ε^2 + a7 ε^3

G = %

>In the above, *u’’’* and *u’’* are equivalent to using different formulae. Therefore, below *u’’’-u’’* is equivalent to zero. In the above, altering u, *u’’’- u’’* is described using only *v*. We set *u’’’- u’’* equal to G that is equivalent to zero. For obtaining coefficients a1-a7 in *u’* in (5.5):

In[34]:=Coefficient[G, ε^3]

% /. v -> 0

c7 = %

c6 = Coefficient[G, v ε^2]

c5 = Coefficient[G, v^2 ε]

Coefficient[G, v^3 ]

% /. ε -> 0

c4 = %

Coefficient[G, ε^2]

% /. v -> 0

c3 = %

c2 = Simplify [Coefficient [G, v ε]]

Coefficient [G, v^2]

% /. ε -> 0

c1 = %

In [55]: =Solve [c1 == 0, a1]

Out [56]: {{a1 -> 906.143}}

In [57]: = {{a1 = 906.1426985058247`}}

In [58]: = Solve [c2 == 0, a2]

Out [59]: {{a2 -> -4.17119}}

In [60]=: {{a2 = -4.171188789989224`}}

In [61]=: Solve [c3 == 0, a3]

Out [61]: {{a3 -> 0. `}}

In [62]: = {{a3 = 0. `}}

In [63]: = Solve[c4 == 0, a4]

Out[63]: {{a4 -> 3.15453*10^6}}

In[64]:= {{a4 = 3.154534012168764`*^6}}

In[65]:= Solve[c5 == 0, a5]

Out[65]: {{a5 -> -23711.1}}

In[66]:= {{a5 = -23711.097334021597`}}

In[67]:= Solve[c6 == 0, a6]

Out[67]: {{a6 -> 38.0354}}

In[68]:= {{a6 = 38.035350136964496`}}

In[69] := Solve[c7 == 0, a7]

Out[69]: {{a7 -> 0.`}}

In[70] :={{a7 = 0}}

>Accordingly, *u* is obtained using the coefficients *a_1_-a_7_*:

In[71] :=

u = a1 v^2 + a2 v ε + a3 ε^2 + a4 v^3 + a5 ε v^2 + a6 v ε^2 + a7 ε^3

Out[71]: 0. - 4.17119 e v + 38.0354 e^2 v + 906.143 v^2 - 23711.1 e v^2 + 3.15453*10^6 v^3

> *v’* (*v’’*) is obtained using *u*:

In[72] := v'' /. u -> -4.171188789989224` e v + 38.035350136964496` e^2 v + 906.1426985058247` v^2 - 23711.097334021597` e v^2 + 3.154534012168764`*^6 v^3

> To neglect small items of high-order,

In[73] := Expand[%]

% /. ε^2 u^2 -> 0 /. ε^3 u v^2 -> 0 /. ε^2 u v^2 -> 0 /. ε^2 u v^2 -> 0 /. ε u^2 v -> 0 /. ε^2 u v -> 0 /. ε u v^2 -> 0 /. u^4 -> 0 /. u^5 -> 0 /. u^6 -> 0 /. ε^2 u^3 -> 0 /. ε u^3 -> 0 /. ε^2 u^7 -> 0 /. ε^3 u^7 -> 0 /. ε u^7 -> 0 /. ε^3 u^3 -> 0 /. u^7 -> 0 /. u^4 -> 0 /. ε^4 -> 0 /. ε^5 -> 0 /. ε^6 -> 0 /. ε^3 u -> 0 /. ε^3 u^2 -> 0 /. ε^7 -> 0 /. v^4 -> 0 /. ε v^3 -> 0 /. ε^2 v^2 -> 0 /. ε^3 v -> 0 /. v^5 -> 0 /. v^6 -> 0 /. ε^3 v^3 -> 0 /. ε^2 v^3 -> 0 /. ε^3 v^2 -> 0

>Output is given by:

Out[73] = 0. - 0.0195967 v + 1.47712 e v - 8.57158 e^2 v - 229.475 v^2 + 6145.63 e v^2 - 930552. v^3

>Here we obtain the stability equation:

>Here o(4) is Landau symbol. Further, setting the right hand of above equal to zero gives;

In[74]:= Solve[-0.019596689731077248` v + 1.477119837741454` e v - 8.571578546078044` e^2 v - 229.47456034957597` v^2 + 6145.626130329382` e v^2 - 930551.7602919037` v^3 == 0, v]

Out[74]=

{{v -> 0.}, {v -> 7.03382*10^-47 (-1.75296*10^42 + 4.69466*10^43 e - 7.8396*10^13 Sqrt[-1.92598*10^56 + 2.54236*10^58 e + 5.56735*10^58 e^2])}, {v -> 7.03382*10^-47 (-1.75296*10^42 + 4.69466*10^43 e + 7.8396*10^13 Sqrt[-1.92598*10^56 + 2.54236*10^58 e + 5.56735*10^58 e^2])}}

>Here the three solution of [74] are given. In addition, setting the root component in the above equation:

In[77] :=

Solve[-1.9259750400082797`*^56 + 2.5423632794192355`*^58 e + 5.567349397506601`*^58 e^2 == 0, e]

Out[77] = {{e -> -0.46411}, {e -> 0.00745386}}

Therefore, the stability equation bifurcates with respect to the value of *e* When *e* > -0.00580481= *e_1_*, because the root component is positive, the stability equation has three real solutions, indicating that the fluctuation has two possible amplitudes other than zero. On the other hand, when *e* ≤ -0.00580481~0, the stability equation has zero solutions. Thus, the stability equation bifurcates around the critical concentration, *p_c_* ~ 0.8.

**Supporting Information Legends**

This supporting information is the detailed explanation of the calculation using Mathematica 8 (Wolfram Research, Inc., Champaign, IL) in the paragraph “Evaluation of the stability of the model around the equilibrium state” in the result section.
